# The Emergence of the Dose–Response Concept in Biology and Medicine

**DOI:** 10.3390/ijms17122034

**Published:** 2016-12-05

**Authors:** Edward J. Calabrese

**Affiliations:** School of Public Health & Health Sciences, Environmental Health Sciences, Morrill I-N344, University of Massachusetts, Amherst, MA 01003, USA; edwardc@schoolph.umass.edu; Tel.: +1-413-545-3164; Fax: +1-413-545-4692

**Keywords:** hormesis, dose–response, biphasic, linear non-threshold, threshold, adaptive response, history of science

## Abstract

A historical assessment of the origin of the dose–response in modern toxicology and its integration as a central concept in biology and medicine is presented. This article provides an overview of how the threshold, linear and biphasic (i.e., hormetic) dose–response models emerged in the late 19th and early 20th centuries and competed for acceptance and dominance. Particular attention is directed to the hormetic model for which a general description and evaluation is provided, including its historical basis, and how it was marginalized by the medical and pharmacology communities in the early decades of the 20th century.

## 1. Introduction

The dose–response relationship is a central concept in many biological disciplines, but especially in pharmacology, toxicology and risk assessment. Despite its centrality in the biological sciences, the origins of dose–response concepts and models remain underexplored and underappreciated. Nonetheless, the area of dose–response, especially with respect to low dose treatment effects, remains highly controversial and unresolved. This confusion may be highlighted with the ongoing debate over the use of the linear-no-threshold dose–response model for cancer risk assessment for ionizing radiation and chemical carcinogens. In 2015, the US Nuclear Regulatory Commission called for a national debate over whether the linear-non-threshold (LNT) model for radiation risk assessment should be replaced by the hormesis dose response [1]. While the current issue is designed to assess in detail aspects of the hormetic dose–response relationship, it was deemed of value to explore the historical foundations of the hormetic dose–response within a broader context, that is, in comparison to the two other leading dose–response models, the threshold and LNT models. The reader may find it surprising to learn that controversy associated with these dose–response models is not a new feature but one that started from the beginning of experimental research, involving each of the major dose–response models, continuing to the present. Thus, while specific controversial issues associated with dose–response models have often changed over generations, the area of dose–response is an active research zone and it is expected that new insights, perspectives and judgments may emerge, with profound medical, public health and regulatory implications. This article provides the historical setting from which new research findings on dose–response models and their underlying mechanisms will emerge to both inform and possibly challenge the status quo.

## 2. The Birth of Dose Response Modernity

While debates have long persisted over the validity and use of the threshold and linear-non-threshold (LNT) dose–response relationships, the first research-based insights into the nature of the dose–response emerged from domain of microbiology, centering on the landmark works in the latter part of the 19th century of Joseph Lister concerning aseptic surgery and the findings of Robert Koch about how to efficiently kill anthrax spores. It is generally recognized that Robert Koch (1881) [2] initiated the process of assessing the bactericidal effects of numerous chemical disinfectants via the use of pure cultures of bacteria. His initial approach was to assess the survival of emulsions of anthrax spores that were dried upon silk threads. He would determine the time of spore survival in solutions of then known disinfectants. The approach of Koch would be progressively modified resulting in more reliable microbiological methods [3]. It was therefore Koch who published the first systematic findings on the killing of anthrax spores with the then popular biocide, carbolic acid, which had been first used by Lister in the process of formulating what became aseptic surgery.

While Koch started the research on disinfectant potency and dose–response, he inspired the actions of many others. One particular group led by Kronig and Paul [4] provided an assessment of disinfectant properties via the use of well-defined microorganisms, the use of numerous disinfectants and all within the framework of a broad range of concentrations. Their efforts revealed a logarithmic relationship between the numbers of bacteria surviving the chemical disinfection treatment and survival times, an observation confirmed by others [3,5]. Of importance to the dose–response debate was that Chick [3] noted that the dose-time-response was very similar to a first order reaction, which she called a unimolecular reaction. Of further significance was that this description is based on the Law of Mass Action, wherein the velocity of the reaction is proportional to the active mass of reacting agent present at that time. In effect, Chick would claim that the velocity of disinfection at any instant is proportional to the number (or weight) of living bacteria present.

**Figure 1:**
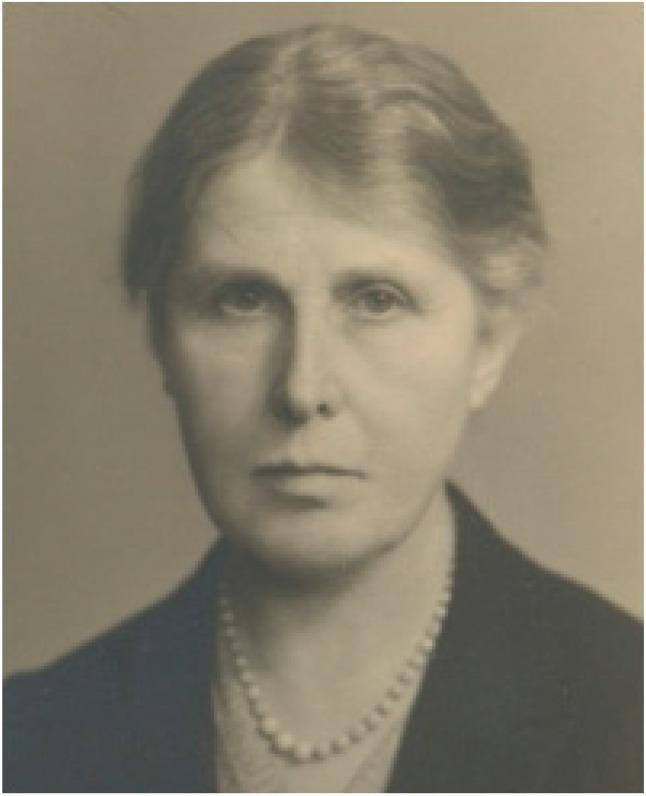
Harriette Chick (1875–1977) [16].

The thinking of Chick was important, not only for its application to disinfection practices at the community level, but also because of its capacity to be generalized to a broad range of agents having environmental relevance, including the then emerging field of radiation biology (Figure 1). For example, Blau and Altenburger [6], expanding the perspective of Chick, found that the destruction of microorganisms by X-rays resulted in unimolecular dose–response curves. Based upon such observations, these authors concluded that the death of cells was mediated via one or at most a few quanta of energy. Such efforts provided the experimental and intellectual framework that was adopted by leading physicists for radiation target theory [7,8,9,10], which was subsequently applied to the newly emerging X-ray mutational data of Muller [11] and Timoféeff-Ressovsky et al. [12] leading to the LNT-single hit theory for genomic mutations, an hypothesis later generalized for radiation and chemically induced cancers [13]. Thus, the theoretical basis of radiation dose–response and the LNT-single hit theory evolved directly from the work of Chick on chemical disinfection. As noted by Packard [14] and Clark [15], the radiation and chemical dose–response controversies are similar and depend upon the need to resolve similar biological/toxicological issues.

The unimolecular hypothesis of Chick created considerable debate in the microbiological literature for the next several decades as seen initially in the reports of Arrhenius [17], Eijkman [18], Hewlett [19] and Reichel [20]. Such reports were followed by those of Loeb and Northrop [21], Brooks [22], Peters [23], Smith [24,25], Shackell [26], and Shackell et al. [27]. These discussions and criticisms eventually coalesced in the writings of Buchanan and Fulmer [28] who noted: (a) A lack of correspondence of the experimental evidence with the theoretical curve at the beginning of the experiment. They were critical of the Chick findings since such studies had inadequate data at short intervals at the temporal start of the experiments. The critics concluded that Chick′s findings simply could not provide a proper assessment of the distribution of susceptibility in the population of bacteria. This was a serious criticism since it undercut predictions at the low end of the distribution; (b) There was also a lack of association of the actual survivors′ curve with the logarithmic curve in the later part of the response curve. For example, when the values of the velocity constant are small they tend to decrease rather than remain constant as predicted; (c) There were also scientific issues with the assumption of uniform susceptibility. Substantial data indicate considerable variability in susceptibility [29,30]. Furthermore, the ratio of susceptible to non-susceptible cell is not constant, which should be the case if the unimolecular model were correct and (d) There were also problems related to providing a theoretical basis for interpreting cell death via a unimolecular reaction.

These criticisms would lead to a general rejection of the unimolecular dose–response concept within most areas of the biological sciences, with the major exception of radiation biology, with its particular focus of the induction of mutations by ionizing radiation. The unimolecular dose–response model was therefore widely outcompeted by the characteristic dose–response theory. This dose–response theory would be built upon an estimation of the distribution of individual variation within the population with regard to susceptibility to toxic substances, including chemical and physical agents.

The most important and penetrating development of the characteristic dose–response was published by Trevan [31]. This work was based on a strong dissatisfaction with predictions such as those derived from the unimolecular approach to estimate minimal effective or toxic doses. He argued that this approach should be replaced with a method that estimates the central tendency of the group response. This concept led Trevan to derive the concept and terminology of the lethal dose 50 (LD_50_) [31], which is lethal to 50% of the exposed population. He also created the term “characteristic” to describe the dose–response representing the percentage of response (e.g., mortality or other biological responses of interest or concern) induced by a range of doses of a drug, industrial agent or radiation on biological models. Trevan then provided the statistical basis for the key practical issues of sample size and statistical power. A strength of the Trevan proposal was that it could provide a statistical vehicle to simulate a response descriptor that could be reliably determined, thereby offering a powerful incentive for additional research.

The leadership of Trevan was extended by the collective efforts of Bliss and Gaddum who would integrate the concepts of Trevan into the major biological disciplines. For example, Bliss published a “how to” statistical road map for dose–response assessment for many key biological disciplines of the 1930s–1960s, including entomology, microbiology, physiology, pharmacology, and toxicology [32,33,34,35,36,37,38,39,40,41]. The writings of Bliss and Gaddum [42,43] became the standard and would be used as basic instructional tools in the education and training of generations of biological researchers and, in many respects, remain so today.

The impact of these biostatistical leaders of the earlier/mid decades of the 20th century was profound. They also provided the foundation for follow up work by Finney [44,45,46] on the development of probit analysis, with its important applications to the field of toxicology. In fact, it can be seen that such thinking was having a significant impact on the then nascent field of cancer risk assessment with its applications in the classic paper of Bryan and Shimkin [47]. This paper evaluated the nature of the dose–response relationship for various chemical carcinogens over a wide range of exposures. It is also ironic that the authors of this paper constrained the dose–response model to estimate only tumor increases, thereby obscuring apparent hormetic effects assuming such responses reflect variability rather than representative treatment effects. Two decades later, the probit analysis approach of Finney would provide the foundation of the influential Mantel–Bryan model [48] for low dose cancer risk assessment as is typically applied when extrapolating far beyond the observable empirical data to very low risks in the one in a million and lower zones for lifetime risks.

In setting the context of the history, documentation and applications of dose–responses in the 20th century, I therefore find that two theories dominated the mainstream scientific community era. These were the unimolecular and the characteristic models. While the unimolecular model was initially more powerful in its appeal and applications, it would be challenged seriously by the characteristic model advocates and lose influence. There were also attempts to broaden and reformulate the characteristic dose–response concept as one describing biochemical processes that mediated inter-individual variation. Moreover, this transformation acquired multiple mathematical forms even down to the present time as seen in various hit and stage theories of carcinogenesis.

Other biostatistical approaches were also developed, such as the logistic method [49,50,51]. For example, the use of logits with quantal data was founded on the assumption that the logarithms of the individual doses were distributed in a complex curve slightly different than that upon which the probit model was based. In fact, Emmens [52] attempted to account for the dose–response curve for mortality as an example using the logistic approach. He further argued that if the concept of tolerance were abandoned due to theoretical assumptions, then the law of chance would favor the use of logits. This view was formulated earlier by Yule [53] in a more fundamental manner, using a random hit theory dose–response method, with the dose–response offering similar features to that seen with the probit curve method.

**Figure 2:**
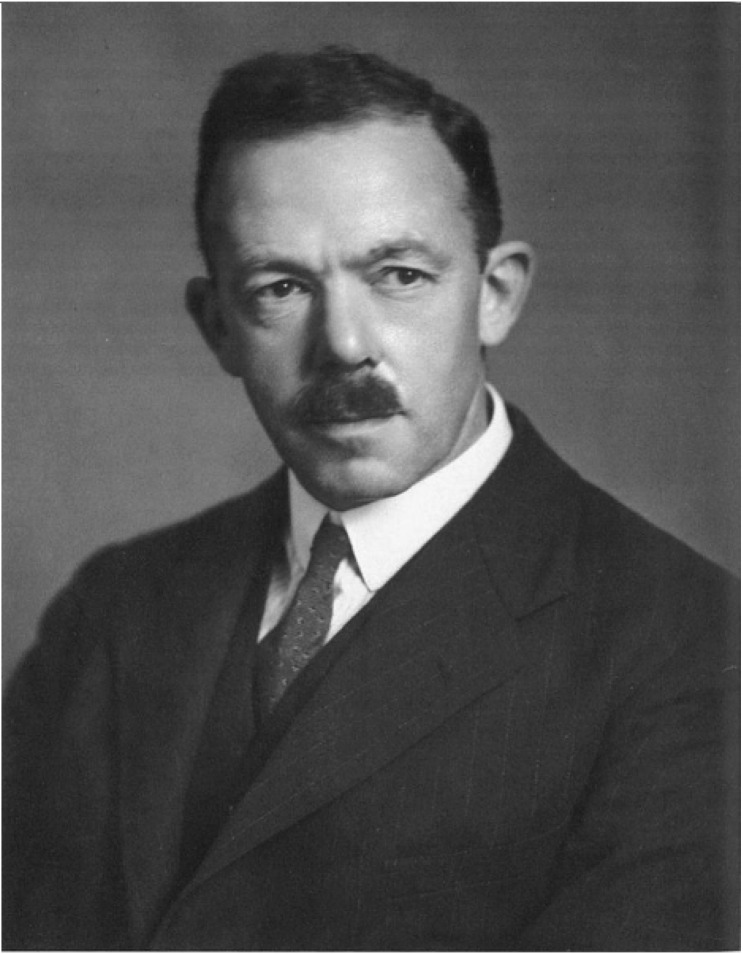
Alfred J. Clark (1885–1941) [54].

The issue of dose–response acceptance was important as it became an object of considerable focus in the influential text by Alfred J. Clark entitled *Handbook of Experimental Pharmacology* in 1937 [15] (Figure 2). This text was critical of the unimolecular theory while providing support for the characteristic curve model, including detailed explanations concerning how it could be integrated into new developments reported with pharmacokinetic processes. However, even in Clark’s extensive criticism of the unimolecular dose–response model, he was very respectful as seen in the comment that “it is obvious that a physico-chemical theory (i.e., unimolecular theory) regarding the mode of action of drugs, which has received the support of Arrhenius must be considered carefully”. The same type of respectful deference was not shown to Schulz and his biphasic dose–response (to be discussed immediately below), rather, just the opposite. Of course, Arrhenius was a Nobel Prize recipient and chair of the Nobel Prize awarding committee.

## 3. The Forgotten Dose–Response Model: Biphasic Dose Response

### 3.1. Hugo Schulz: The Discovery of Hormesis

We can thus see that dose–response debate and controversy did not start with the onset of the environmental revolution of the 1970s and the issues over how to estimate the risk of carcinogens at very low doses. In fact, the above discussion demonstrates that two groups of mainstream biological/biomedical scientists had explored and debated these issues for the previous half century prior to the so-called modern dose–response era. Of particular relevance to the present paper is that it was within this dynamic intellectual environment that the issue of the hermetic–biphasic dose–response emerged and evolved. However, one thing is obvious right from the start: the unimolecular and the characteristic dose–response concepts originated within two opposing camps of mainstream scientists and, as a result, their conflicts would be followed, debated and respected. What would become the hormetic dose–response originated in an entirely different manner, emerging from the long-standing dispute between traditional medicine and homeopathy. Since the hormetic dose–response was claimed by its discoverer, Hugo Schulz, to provide the explanatory principle of homeopathy, Schulz’s biphasic dose–response model and himself became the object of much criticism from both dose–response camps, but especially by the characteristic curve model group, as highlighted in the very influential writings of Clark.

**Figure 3:**
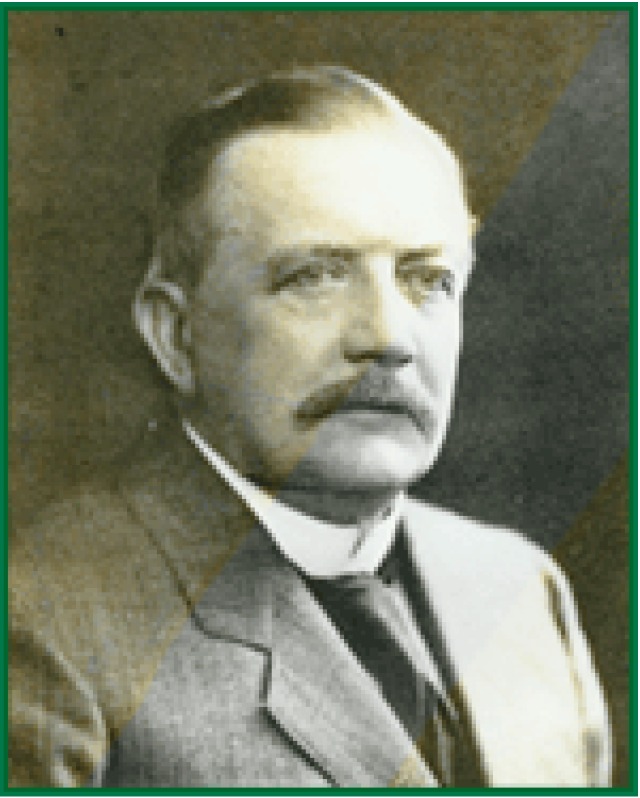
Hugo Schulz (1853–1932) [55].

Despite its characterization here as the “forgotten dose–response”, the biphasic dose–response relationship was the first dose–response model to be experimentally formulated. The initial data underlying this development were generated by Hugo Schulz (1853–1932), a physician who was well trained in pharmacology and toxicology (Figure 3). This research was undertaken at the University of Greifswald in northern Germany, probably in late 1883, with his first presentation on this topic to the scientific community occurring at a local meeting of Greifswald Medical Society in 1884. Schulz had done extensive laboratory research assessing the effects of various chemical disinfectants on the survival and metabolism of yeasts [53]. In fact, he was a young contemporary of Robert Koch who was doing similar research but with bacteria. Koch would soon become famous for his discoveries relating to the life cycle of anthrax. Koch would go on to create a powerful research program in basic and public health microbiology, with three of the first seven Nobel Prize winners in Biology and Medicine being from Koch′s laboratory, including himself.

The scientific path of Schulz would be different. In his studies on the effects of multiple chemical disinfectants, Schulz incorporated a broad dose–response feature, a time component as well as a metabolic measure along with the standard mortality endpoint used by others. In fact, Schulz′s study designs were more sophisticated and robust than the future Nobel Prize winner Koch. As a result, Schulz observed an unexpected biphasic dose–response in which high doses were toxic and suppressed metabolism, while the opposite seemed to occur at low doses. This troubled Schulz, making him think that he must have had some type of methodological error in his experiments. However, copious replications and other assessments gave him high confidence that his findings were real and reproducible as revealed in his reflective comment below [56]:

“Since it could be foreseen that experiments on fermentation and putrescence in an institute of pathology would offer particularly good prospects for vigorous growth, I occupied myself as well as possible, in accordance with the state of our knowledge at the time, with this area. Sometimes, when working with substances that needed to be examined for their effectiveness in comparison to the inducers of yeast fermentation, initially working together with my assistant, Gottfried Hoffmann, I found in formic acid and also in other substances the marvelous occurrence that if I got below their indifference point i.e., if, for example, I worked with less formic acid than was required in order to halt the appearance of its anti-fermentive property, that all at once the carbon dioxide production became distinctly higher than in the controls processed without the formic acid addition. I first thought, as is obvious, that there had been some kind of experimental or observation error. But the appearance of the overproduction continually repeated itself under the same conditions. First I did not know how to deal with it, and in any event at that time still did not realize that I had experimentally proved the first theorem of Arndt’s fundamental law of biology.”

These findings should have been of considerable interest to Robert Koch and Joseph Lister, amongst others. However, something happened during the next step of hypothesis development that changed the course of Schulz′s professional life and the development of dose–response theory and practice down to the present time. The biphasic dose–response observations soon became integrated into a general biologically based dose–response framework by Schulz and his colleague at Greifswald, Rudolph Arndt. So convinced of the correctness and generality of their conceptual dose–response model, the creators designated their model a biological law, called Arndtt–Schulz Law. A protégé of Robert Koch, Fernindand Hueppe, generalized their findings to bacteria, strangely renaming the phenomenon Hueppe′s Rule, while at the same time acknowledging the primacy of Schulz [57].

In retrospect, this dose–response theory of Schulz and Arndt was conceptual and mostly intuitive, with the data supporting it limited but acceptable on their own merits. However, it was the integration across diverse studies and the interpretation of the data that were problematic. More specifically, Schulz was interested both in chemical disinfection and in testing features of homeopathy. With respect to the latter, Schulz learned of an 1884 study in which the homeopathic preparation called veratrine was used to successfully treat gastroenteritis in humans [58]. This intrigued Schulz who went to Koch to obtain a pure culture of the bacterium causing the disease. Schulz wanted to test whether the veratine could actually kill the causative agent, and thereby obtain insight into the possible mechanism of the homeopathic treatment. However, regardless of the dose, veratine was unable to kill this disease-causing agent. While some scientists may have questioned the reliability of the veratine findings of Bloedau [58], Schulz and Arndt did not. In light of Schulz′s other research with yeast, Schulz and Arndt came to the view that veratrine was an effective agent against gastroenteritis but it did so, not by killing the bacteria itself, but by enhancing the adaptive capacity of the human to fight off the infection. They came to this conclusion by linking the yeast findings that indicated that the large number of chemical disinfectants tested acted differently at low dose, enhancing survival. Thus, Arndt and Schulz developed the hypothesis that most agents act biphasically and that they induce adaptive survival enhancing responses at low doses. They then applied this concept not only to veratine but also to homeopathic drugs in general. It was within this context that they derived the perspective that they had discovered the underlying explanatory principle of homeopathy. It was with the public announcement of this theory that the problems of Schulz and this biphasic dose–response model, and eventually the term hormesis, would begin.

The problem for Schulz and his model was that homeopathy and traditional medicine were in a major and longstanding conflict over which medical practice would come to dominate society [59,60]. There was much animosity over the issue. By linking his biphasic dose–response theory to homeopathy, Schulz ensured that it would become the object of profound criticism and would be rejected by the biomedical community. This should not have been hard to predict.

The biomedical community would go to great lengths to marginalize Schulz and his dose–response model. This started right away as is evident in the contemporary literature and from multiple perspectives. The contemporary research rival Hueppe argued that the findings of Schulz should not be rejected even though he made the profound error of associating it with homeopathy [57]. However, most critics were not so sympathetic. This may be best seen in the copious writings of Clark, who became a leading critic. Clark did his best to link Schulz with the high dilution Hahnemann wing of homeopathy (see Calabrese [61], Tables 1–3 for numerous examples of such efforts by Clark). This was done to both discredit Schulz and his dose–response, even though Schulz was adamant in his writings that he did not support the high dilution views of followers of Hahnemann who argued that biological effects could occur below Avagadro′s number [62]. For example, Clark would write that the Arndt–Schulz dose–response law was “in accord with homeopathic doctrines”, implying that it derived its foundation from a homeopathic rather than a biological/toxicological traditional. Clark would also state that the Arndt–Schulz Law “is obviously untrue in the case of most drugs that have been studied carefully”, yet failing to provide the documentation to support such a conclusion.

The statements of Clark were also inconsistent with a substantial series of independent reports in the biological literature that were strongly supportive of the Schulz dose–response model [63,64,65,66,67]. However, the views of Clark would carry the day, as Clark and many of his colleagues in the British pharmacological community were prominent leaders in the domain of traditional medicine and extremely accomplished researchers in their own right. When matched against such a profoundly accomplished and committed opposition, Schulz would have little chance to influence the direction of the field. Furthermore, Schulz′s career was so affected that he was unable to consider moving to more prestigious academic institutions, as was commonly done during that era, being relegated to Greifswald for his entire professional life. The travails of Schulz and his biphasic dose–response were highlighted in a sympathetic memorializing of his life by a colleague, who recounted the challenges and unfair and often deceitful characterizations by otherwise leading scientists during that era, all in an effort to destroy homeopathy, making Schulz and the hormesis concept what today one might call “collateral damage” [68].

### 3.2. Generalizing the Biphasic Dose Response

Despite the profound difficulties that Schulz endured, many researchers published findings of biphasic dose–response relationships, especially in the area of plants, microbiology and entomology with both chemicals and radiation. The findings of Schulz stimulated numerous doctoral dissertations [69,70,71,72,73] that generally confirmed and extended his findings. Numerous other dissertations addressing the stimulation of bacterial growth by low doses of toxic agents were conducted under the direction of Charles Winslow, the Yale University professor of bacteriology and longtime editor-in-chief of the *Journal of Bacteriology* and later the *American Journal of Public Health*. For example, Hotchkiss [74,75] provided a comprehensive survey of the stimulatory and inhibitory/toxic effects of both minerals and toxic metals on Escherichia coli (*E. coli*). Of particular interest was that the agents were usually tested over a broad concentration range with six or more doses. Most of the agents tested displayed low dose stimulation, including the salts of lead, mercury, nickel, tin, titanium and strontium.

The work of Hotchkiss revealed that the stimulatory response was strongly influenced by the nature and the quality of the study design. Experiments with large numbers of doses, especially with multiple treatments below the toxic threshold, displayed consistent stimulatory responses in this low dose zone. The median maximum stimulatory responses were modest, being about 50% greater than the controls, while the stimulatory range was more variable, extending from 2- to 100-fold below the threshold, with an average of about 50-fold. The work of Hotchkiss was to stimulate a long line of subsequent graduate students at Yale University to extend these findings [63]. Furthermore, the study design features implemented by Hotchkiss under the direction of Winslow created a type of research standard for the assessment hormetic-like biphasic dose–responses in terms of number of doses, dose range and spacing, and replications. This research was significant as it led to the general recognition by the 1930s that disinfectants display a biphasic dose–response, with knowledge of this phenomenon becoming so recognized and accepted that it became incorporated into standard microbiological texts during the middle decades of the 20th century [76,77,78].

The biphasic effects of disinfectants on bacteria were paralleled with similar findings concerning the effects of various toxic inorganic agents on the ammonification, nitrification and nitrogen-fixation in soil by various bacterial species. This research was initially studied in 1913 by the well-known bacteriologist Lipman [79] from the University of California at Berkeley who was interested in assessing the impact of large quantities of waste alkali on the capacity of soil bacteria to perform ammonification and nitrification. Low dose stimulation responses by bacterial ammonifiers were commonly observed. At the same time, Greaves [80,81,82] revealed that various chemical insecticides likewise induced hormetic-like biphasic dose–responses on the bacterial ammonification process. Greaves was unusual in his study designs, using from 20 to 30 concentrations over a wide concentration range. The findings of Greaves were noted for their consistency of responses between replicate studies. Similar findings were also reported for various uranium compounds, again with strong study designs [83].

Lipmann and his colleagues would be the first group to apply the concept of hormesis to risk assessment in a legal case dealing with smelter works in California. They presented data that low doses of toxic metals such as arsenic and lead stimulated rather than inhibited plant growth. See Calabrese [84] for a detailed description and assessment of this case.

The story of hormetic-like biphasic dose–responses just briefly summarized for bacteria also occurred with fungi, yeast, insects and plants using various chemicals and radiation as inducing agents during the early decades of the 20th century. The findings were often reported by experienced investigators, typically with adequate to strong study designs and published in the leading journals of that era. However, these findings were never adequately summarized and integrated during the 20th century. It was only during the resurgence of the hormesis concept at the very end of the 20th century that this extensive published network of early historical findings on hormetic dose responses was revealed to contemporary biological and biomedical scientists. Of further note was that a German language journal *Cell Stimulation* was published during the 1920s. Likewise, an academic journal-like publication called the *Stimulation Newsletter* was published that addressed the capacity of radiation to induce stimulation in plant growth. The history of these activities has been reconstructed and published in an entire issue of the journal *Human and Experimental Toxicology* [63,64,65,66,67].

### 3.3. Debates over Acceptance and Biological Meaning

**Figure 4:**
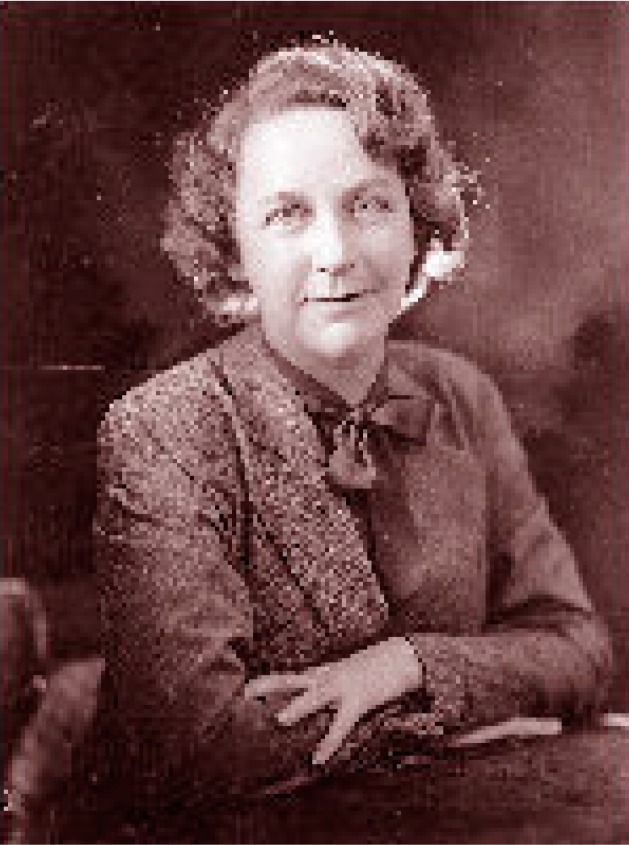
Sara Branham (Matthews) (1888–1962) [88].

These findings were to force some investigators to struggle with the actual definition of the hormetic dose–response. Perhaps the most significant theoretical debate centered on whether the low dose stimulation was a direct one or an overcompensation to a disruption in homeostasis, that is, some minor degree of toxicity. A number of extremely well designed and conducted studies with different biological models and inducing agents provided convincing evidence that a low dose stimulation may occur as a result of an overcompensation to an induced initial toxicity. Of particular note were findings of Sarah Branham [85] (Figure 4) of the University of Rochester who sought to provide a very explicit, detailed and advanced replication of the original findings of Schulz that stimulated interest in the biphasic dose–response concept. Her findings were striking in that she not only reported that low concentrations of numerous chemical disinfectants stimulated the growth of yeast colonies but also did so in a manner that clearly involved an overcompensation to an initial toxic response. This type of dose-time–response was also reported by others such as Professor Elizabeth Smith [86] of the University of Wisconsin who reported that UV radiation induced a biphasic dose–response for mycelium growth in which the stimulatory response occurred only after the UV-induced initial damage with a rebound stimulation reflecting the overcompensation response. Large numbers of similar overcompensation stimulation dose–responses have now been reported and summarized [87].

Of significance was that the reporting of a low dose stimulation after an initial toxicity was viewed by some as a refutation of the hormesis hypothesis. This was particularly the case in the area of radiation biology. For example, while Manfried Fraenkel argued that low doses of ionizing radiation can stimulate biological processes by a direct positive effect [89], Holzknecht and Pordes rejected the possibility of a direct stimulatory response without an initial induced damage [89]. The confusion over whether the Arndt–Schulz Law was the result of a direct response or a phenomenon following a response to damage became an important conceptual battle that was still evident several decades later. This dispute was important since it attracted many leading researchers in the field of radiation and its medical applications such as Holzknechzt, a former colleague of Roentgen and the person recognized as having created the first method of quantifying X-ray exposure. He was also the first European professor of medical roetgenology [89]. The lack of both resolution and understanding of the concept of hormesis also eroded its acceptance as the rapidly maturing field of radiation biology/medicine entered the 1940s. This issue was highlighted when the prestigious Harvard professor and first director of the Division of Biology and Medicine at the US Atomic Energy Commission, Shields Warren [90], continued to promote the concept of Holzknecht and Pordes with comments that the “assumption that small doses of X-ray or radiation are stimulatory (the Arndt–Schulz “Law”) is invalid. The slight evidences of proliferative activities offered as evidence by the proponents of this hypothesis are in fact only reparative responses to the injury that has been done”. Warren would continue to provide considerable leadership to the field, serving on the first US NAS BEAR Committee in 1955–1956, being the chair of the Pathology Panel and a member of the Genetics Panel that recommended a switch from a threshold to a linear dose–response model for risk assessment purposes.

The rejection of the Arndt–Schulz Law by key leaders in the radiation community such as Shields Warren over the fact that radiation often induced stimulation via an overcompensation response following damage was a significant judgment leading to the continued marginalization of the hormesis concept. These leaders failed to grasp that radiation and chemicals had the capacity to induce stimulatory responses at low doses via either direct (as established below) or overcompensation processes. They also failed to recognize that the quantitative features of these dose–responses were similar regardless of the means of stimulation induction. In fact, it is particularly ironic that now, more than seven decades following such marginalizing judgments, the definition of hormesis incorporates the overcompensation response following a disruption in homeostasis concept along with a direct stimulation component [91]. This overcompensation stimulation concept of hormesis is in fact the same definition that was rejected by leaders such as Holzknecht and Warren. It therefore seems that these early leaders within the radiation community had derived a clear scientific understanding of the overcompensation concept but marginalized it to the point that it was not considered a significant biological phenomenon. In fact, the overcompensation stimulation concept of hormesis, which was rejected due to its lack of apparent biological relevance, evolved into a modern biological/toxicological hormetic mechanism theory by Stebbing [92,93] by the late 1970s involving various feedback compensatory mechanisms.

Even when the stimulatory response was the apparent result of a direct stimulatory response, it was often not considered of particular importance. For example, the widely cited publication of Marshall and Hrenoff [94] emphasized that the stimulatory response to disinfectants “is frequently of no practical value”. The inclusion of the stimulatory dose range for agents such as disinfectants was for illustration of the completeness of the entire dose–response spectrum rather than for its biological significance.

Even the well-known bacteriologist Otto Rahn [95] modeled the hormetic-biphasic dose–response. He noted that this model was in fact widespread and generalizable. Importantly, he offered a mechanism, involving an enzymatic explanation for the low dose stimulatory response. Using an example of the effect of arsenic on zymase activity, he suggested that the toxic agent most likely acts as a catalyst, enhancing enzyme activity along with enzyme degradation. He proposed that there was a shifting of the optimum enzyme activity with time from higher to lower concentrations of the toxic agent. While Rahn offered an early biostatistical-model based framework to assess biphasic dose–responses, this work, like that of many other investigators, failed to emerge and thrive during the first half of the 20th century, in contrast to its dose–response rivals.

While some of the blame for the failure of the hormesis concept to thrive can be placed on the actions of prominent scientists such as Clark [96], a substantial contributory factor to the early demise of the biphasic dose–response was due to the lack of leadership and organizational activity of prominent researchers in this area. Further, a detailed assessment of essentially all the leading early hermetic-biphasic dose–response researchers has revealed that most redirected their scientific careers to governmental service or academic administration or other divergent but compelling research activities [97]. In many ways, the hormetic dose–response failed to thrive during this period due to a combination of factors, all of which converged, leading to its continuing marginalization and the exclusion of these findings from the mainstream of science and regulatory application.

The research on hormetic-like biphasic dose–response relationships in the first half of the 20th century was therefore reasonably substantial, competently conducted and fairly general, affecting a wide range of biological models, endpoints and agents. It also became clear that the biphasic dose–response could occur via a direct stimulation or via an overcompensation to an initial disruption of homeostasis. Despite these general findings, the hormesis concept kept being tied to homeopathy due in large part to the work of Schulz, the misrepresentations of Clark, and the need for the homeopathic community to base their therapeutic practices on a well-substantiated hypothesis.

Despite the various struggles encountered by the hormetic dose–response during the first half of the 20th century, a resurgence of interest occurred in this concept toward the end of the 20th century and beginning of the 21st century. Propelling this resurgence was the shift to assess low doses of chemical agents and the use of large scale in vitro testing, which facilitates the use of a larger number of concentrations than typically used in in vivo studies. A third point of dose–response convergence was that hormetic-like biphasic dose–responses were reported very broadly and reproducibly across biological and biomedical subdisciplines, suggesting the widespread generality of the hormetic dose–response relationship [97,98,99,100,101,102]. Finally, if the hormetic dose–response were acknowledged as the preferred dose–response model, it would significantly focus public health investments to more productive areas of societal public health concern.

## Abbreviations

LNT	Linear non-threshold
LD50	Lethal dose 50
